# Rootstock-Mediated Effects on Cabernet Sauvignon Performance: Vine Growth, Berry Ripening, Flavonoids, and Aromatic Profiles

**DOI:** 10.3390/ijms20020401

**Published:** 2019-01-18

**Authors:** Yu Wang, Wei-Kai Chen, Xiao-Tong Gao, Lei He, Xiao-Hui Yang, Fei He, Chang-Qing Duan, Jun Wang

**Affiliations:** 1 Center for Viticulture & Enology, College of Food Science and Nutritional Engineering, China Agricultural University, Beijing 100083, China; wangyu_0919@cau.edu.cn (Y.W.); phyllis4yt@cau.edu.cn (W.-K.C.); xiaotong_gao@cau.edu.cn (X.-T.G.); helei@cau.edu.cn (L.H.); yangxiaohui1128@163.com (X.-H.Y.); wheyfey@cau.edu.cn (F.H.); duanchq@vip.sina.com (C.-Q.D.); 2 Key Laboratory of Viticultural and Enology, Ministry of Agriculture and Rural Affairs, Beijing 100083, China

**Keywords:** Cabernet Sauvignon, rootstock, graft, vine growth, ripening, flavonoids, volatile compounds

## Abstract

Rootstocks are widely used in viticulture due to their resistance to biotic and abiotic stress. Additionally, rootstocks can affect vine growth and berry quality. This study evaluated the effects of eight rootstocks (101-14, 110R, 5A, 5BB, Ganzin 1, Harmony, Riparia Gloire, and SO4) on the vine growth, berry ripening, and flavonoids and aromatic profiles of Cabernet Sauvignon in two consecutive seasons (2015–2016). With few exceptions, minor differences were observed among grafted and own-rooted vines. Own-rooted vines produced the least pruning weight but the highest yield. 101-14, 5BB, and SO4 slightly reduced total soluble solids, but increased acidity, showing tendencies for retarding maturation. Ganzin 1 inhibited the accumulation of flavan-3-ols in berry skins. Furthermore, concentrations and proportions of epicatechin-3-*O*-galate were decreased by rootstocks, except for 110R. 5A, Harmony, and Riparia Gloire enhanced flavonol concentrations. SO4 slightly decreased most of the individual anthocyanin concentrations. With respect to volatile compounds, 110R, Riparia Gloire, and SO4 induced reductions in concentrations of total esters, whilst 101-14, Ganzin 1, 110R, and 5BB led to increases in the concentrations of C_13_-norisoprenoids. Therefore, with respect to the negative effects of SO4 on berry ripening and the accumulation of anthocyanin and volatile esters, SO4 is not recommended in practice.

## 1. Introduction

Rootstocks are resistant to various pests and diseases, and are tolerant to different abiotic stress. Therefore, grafting is a technique widely used in viticulture. Many researchers have evaluated the effects of rootstocks on vine growth and fruit composition. However, up to now, no agreements were reached due to the complex interactions among rootstocks, scion cultivars, soil, and climatic conditions. With respect to vine vigor, several previous studies reported significant differences among different grafted vines [1,2,3,4,5,6]. For example, own-rooted ‘Thompson Seedless’ vines produced the lowest pruning weight in comparison with five grafted vines [7]. Cookson et al. [8] revealed that many genes regulating carbohydrate metabolism and sugar transportation were up-regulated by rootstocks. Therefore, the vigorous growth of vines may be affected by rootstocks through modifying the effectiveness of carbon transportation. Nevertheless, Berdeja et al. [9] found that the differences in the pruning weight of ‘Pinot Noir’ between 110R and 125AA were not significant. Besides, Nelson et al. [10] found that rootstocks did not affect leaf area. Several factors may modify the influence of rootstocks on vine vigor. A previous study reported that rootstock effects on pruning weight varied across different scion cultivars [11]. In addition, the influence of rootstocks on pruning weight and canopy size was closely associated with soil water supply, acidity, nitrogen level, and potassium availability [4,12,13,14]. Sufficient nutrient and water in soil may mask the effect of rootstocks on vine growth. There is a close relationship between vine vigor and yield, which determine vine balance together. Rootstocks can affect the biomass allocation between the root system and the shoot, and then modify the biomass distribution between the fruit and the rest of the vine [7]. Several studies documented that rootstocks imparted significant effects on vine yields [2,4,6,15,16]. Variations in shoot length and vine vigor may affect the sunlight exposure during fruit bud differentiation, thus modifying vine yields [14]. By contrast, Wooldridge et al. [5] found that yields of ‘Chardonnay’ and ‘Pinot Noir’ grafted on four rootstocks, respectively, did not statistically differ in six consecutive seasons. Rootstock effects on yield formation may depend on scion cultivars and seasons. In addition, soil type is correlated with the influence of rootstocks on vine yields. Yields of ‘Merlot’ grafted on 110R and 101-14 only significantly differed in shale soil when compared to granite soil [13].

Grape compositions may be affected by rootstocks through several mechanisms. It is widely accepted that rootstocks affect berry composition through modifying the vine growth, which may alter the microclimate of grape clusters. Nelson et al. [10] attributed the differences in anthocyanins between two grafted vines to the variations in the canopy size, which can mediate the sunlight exposure accepted by grape clusters. Besides, several researchers have suggested that rootstocks may alter the fruit ripening rate, and then modify grape compositions, such as sugar, acidity, and so on [3,4,10]. It has been demonstrated that the period between budburst and flowering is much longer in *Vitis berlandieri* compared to *Vitis riparia* and *Vitis rupestris* [17]. In addition, Corso et al. [18] revealed that the differences in the ripening rate between two grafted vines of ‘Cabernet Sauvignon’ were associated with the expression of genes controlling auxin action (ARF and Aux/IAA). Furthermore, different root system architectures can result in variations in the water and/or nutrient capability of different rootstocks [19]. It was reported that rootstocks with *Vitis rupestris* and *Vitis berlandieri* genetic makeup showed a good ability for nutrient uptake [7]. The variations in the nutrient element use efficiency of vines induced by rootstocks also affect grape compositions. For example, juice acidity and pH are largely determined by the content of potassium, which precipitates tartrates. Besides, the nitrogen capability of grapevines plays an important role in vine growth, which is closely related to grape composition, as described above. Several studies have indicated that grape compositions may be regulated by rootstocks through affecting the concentrations of amino acids in grape berries [7,9,19,20]. Jogaiah et al. [7] showed that the accumulation of sugar may be affected by rootstocks through regulating the ratio of arginine to proline in berries. Besides, since several secondary metabolisms, such as phenylpropanoids biosynthesis, are closely associated with the amino acids, variations in amino acids may induce different concentrations of secondary metabolites in berries among different grafted vines. At the molecular level, even though the molecular mechanism underlying the interaction between rootstock and scion remains unclear, several researchers have revealed that rootstocks can mediate scions through the modification of hormones; the regulation of gene expression; and the large-scale movement of proteins, mRNAs, and small RNAs [21]. Jin et al. [20] revealed that the expression of genes related to sugar, amino acids, and flavonoid metabolism in ‘Gold Finger’ berries was largely modified by rootstocks.

Overall, each rootstock should be evaluated for the given cultivar in a specific region due to the complex interactions among rootstocks, scion, soil, and climatic conditions. Besides, previous studies have focused on the influence of rootstocks on vine growth and berry ripening, whereas information on rootstock-mediated effects on secondary metabolites, especially flavonoids and volatile compounds, is very limited. Therefore, this study evaluated the effects of eight common commercial rootstocks with the parental background of *Vitis berlandieri*, *Vitis riparia*, and *Vitis rupestris* on the vine growth, berry ripening, flavonoids, and aroma profiles of ‘Cabernet Sauvignon’, aiming to provide comprehensive information which may assist growers with rootstock decisions.

## 2. Results and Discussion

### 2.1. Meteorological Data

**Figure 1:**
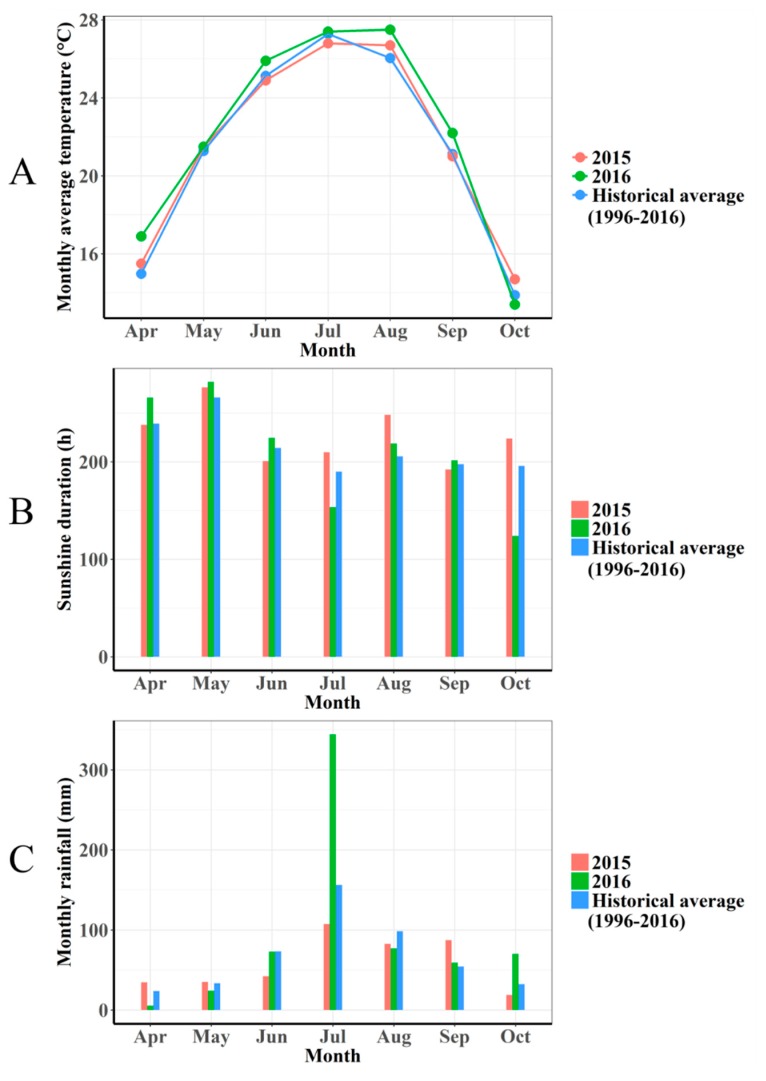
Meteorological data (monthly average temperature (**A**), sunshine duration (**B**), and rainfall (**C**)) of the experimental field from April to October in 2015–2016.

The vineyard site has a continental monsoon type climate. The climate conditions, including temperature, sunshine duration, and rainfall, are summarized in Figure 1. The average temperature during April to September in 2016 was a little higher than that in 2015. The sunshine duration and rainfall in July displayed great differences between the two seasons. To be specific, rainfall in July in 2016 was three-fold higher than that in 2015; consequently, sunshine duration in July in 2016 was distinctly lower than that in 2015. Notably, veraison commences in late July; therefore, climatic differences in July between two seasons could have a great influence on berry ripening and grape compositions.

### 2.2. Effects of Rootstocks on Vine Growth

As shown in Table 1, all the ratios of rootstock to scion diameter in grafted vines were significantly lower than those in own-rooted vines, showing the swelling of the Cabernet Sauvignon scion with respect to the rootstocks at the graft joint. The swelled scion above the graft union was also found in a previous study [7]. Pruning weight is an indicator of shoot vigor [11], and the pruning weight of vines in 2015 was significantly higher than that in 2016, indicating that vines grew mature over the years. Besides, the effects of season on vine pruning weight far outweighed any difference in pruning weight due to the rootstocks. In this study, no significant differences in pruning weight were observed among grafted and own-rooted vines (Table 1), which is in agreement with a previous study [9]. Contrarily, some studies have reported that rootstocks imparted a statistical influence on vine pruning weight [1,2,3,4,5,6]. The vigor control mechanisms of different rootstocks are complex. In addition, rootstock effects on vine pruning weight can be modified by vintage, rootstock genotype, scion cultivars [11,22] and soil condition (water supply, acidity, macro-element level) [4,7,12]. Notably, own-rooted vines produced the lowest pruning weight in this study, and there were tendencies for rootstocks to confer increased vigor on the scion (Table 1). Similar results were reported in the previous study in which Thompson own-rooted vines possessed the least vine vigor (pruning weight) in comparison with five grafted vines [7]. Through transcriptome analysis of the shoot apex collected from the hetero-grafted and auto-grafted Cabernet Sauvignon vines, Cookson et al. [8] revealed that rootstocks triggered the up-regulation, rather than down-regulation, of gene expression in hetero-grafted vines. Besides, many genes from major and minor carbohydrate metabolism and sugar transporters were differentially regulated in hetero-grafted grapevines, which may alter the effectiveness of carbon transportation.

Annual variations dominated the variations in yields; specifically, the average yield in 2016 was significantly higher than that in 2015. Rootstocks had no significant effects on yields (Table 1). In previous studies, there were no consistent conclusions for the influence of rootstocks on yields [1,2,3,5,6,15,16,23]. Rootstocks can give rise to different reactions to yield formation in different types of soil, and it was reported that yields of different grafted vines only significantly differed in shale soil when compared to granite soil [13]. Overall, the influence of rootstocks on yields may depend on seasons, scion cultivars, and soil types. Notably, in this study, own-rooted vines showed the highest yield in comparison with the other graft combinations (Table 1). Keller et al. [11] also found that own-rooted vines produced lower yields than grafted vines in one of three seasons.

The vine yield to pruning weight ratio (crop load) is generally used to determine whether a grapevine is balanced. It was estimated that the crop load of balanced vines ranges between 4 and 10 [17]. In this study, seasonal effects contributed to the dominant variations in crop load, which was significantly higher in 2016 than in 2015 (Table 1). Keller et al. [11] also reported that most of the variations in the yield to pruning weight ratio were attributed to the seasonal effects followed by rootstock effects. In this study, the average vine yield to pruning weight ratio in 2015 was far blow 4, indicating that vines were excessively vigorous due to the young vine age. Despite no significant differences being observed among grafted and own-rooted vines, own-rooted vines with the highest yield and the lowest pruning weight produced the highest crop load in comparison to grafted vines (Table 1). In addition, the vine yield was negatively correlated with the pruning weight (r = −2.45, *p* < 0.001, *n* = 54) in this study, which was also reported in the previous study [15]. Variations in crop load among grafted and own-rooted vines were probably due to the rootstock effects on biomass partitioning between roots and shoots, and then between the fruit and the rest of vine [24]. In addition, water and/or nutrient uptake capability may differ among grafted and own-rooted vines due to different root system architectures, and vine vigor and crop yield may then be modified by rootstocks [19].

### 2.3. Effects of Rootstocks on Berry Physiochemical Parameters

Compared to the own-rooted vines, berry weight was not altered by rootstocks in this study (Table 2), which was in agreement with previous studies [1,2,4,9]. Contrarily, significant differences in berry weight among different grafted vines were also reported [10,22]. The influence of rootstocks on berry weight may be dependent on rootstock genotypes and seasons [3]. The increases in berry weight of ‘Summer Black’ induced by SO4 and 5BB were attributed to the enhanced relative water content. In the present study, slight increases in berry weight were only observed in CS/5BB rather than CS/SO4 (Table 2).

In this study, berries in 2015 accumulated more total soluble solids than berries in 2016. Higher rainfall and lower sunshine duration in July in 2016 compared to 2015 may have delayed berry ripening. Rootstocks did not significantly affect total soluble solids (Table 2), which is in agreement with previous studies [2,5,10,11,15]. However, the significant influence of rootstocks on total soluble solids was also documented [3,4,10,25]. Corso et al. [18] revealed that rootstocks may modify the berry ripening rate through regulating the expression of genes controlling auxin action (ARF and Aux/IAA). In this study, 101-14, 5BB, and SO4 slightly reduced total soluble solids compared to own-rooted vines, which is similar to the results reported by Jin et al. [19]. Besides, the total soluble solids value in CS was higher relative to that in grafted vines, except for CS/Ganzin 1 and CS/Harmony (Table 2). It was reported that 110R with *Vitis berlandieri* genetic makeup tended to retard berry maturation and produce lower total soluble solids when compared with 101-14 (*Vitis riparia* × *Vitis rupestris*) [1,17]. However, in this study, CS/110R accumulated a similar amount of total soluble solids to CS/101-14. In addition, CS/Harmony produced significantly higher total soluble solids in comparison with CS/SO4 (Table 2).

Juice titratable acidity and pH were not affected by either rootstock or season in this study (Table 2). There have been no agreed conclusions about the influence of rootstocks on titratable acidity and pH in previous studies [2,5,10,16,17,19,20,22,26,27]. Since potassium can precipitate tartrates in juice, leading to the reduction of tartaric acid, potassium in juice determines the titratable acidity and pH to a great extent. The alterations of titratable acidity and pH induced by rootstocks were generally observed concomitantly with the modified potassium concentrations led by rootstocks. Potassium uptake capability of grapevine depended on scion cultivars, rootstock genotypes, and soil conditions. Same rootstock may have varied effects on potassium concentrations in berries on different scion cultivars [28]. Besides, variations in the potassium absorption ability of different rootstocks can be attributed to the rootstock genetic origins. It was reported that rootstocks with a *Vitis berlandieri* and/or *Vitis rupestris* genetic background were generally observed for having a good nutrient uptake ability [2,7,29]. The greater availability of potassium from the granite soil may mask the variations in the potassium uptake capability of rootstocks [13]. Overall, in this study, the lack of significant differences in juice titratable acidity and pH among different grafted vines may be attributed to the high potassium availability of the vineyard soil. In addition, CS/101-14, CS/5BB, and CS/SO4 produced a little higher titratable acidity compared to CS (Table 2), which is in agreement with a previous study [19]. Combining the lower concentrations of total soluble solids in CS/101-14, CS/5BB, and CS/SO4 relative to CS, 101-14, 5BB, and SO4 showed tendencies to retard berry maturation in this study.

### 2.4. Effects of Rootstocks on Berry Flavonoids

With few exceptions, minor differences in flavan-3-ol concentrations and proportions in grape skins emerged among grafted and own-rooted vines (Supplementary Figure S2), which is consistent with previous research [4,27,30]. In particular, 101-14, Riparia Gloire, and SO4 had almost no influence on total flavan-3-ol concentrations in the two seasons. Besides, the season factor dominated the effects of 110R, 5A, 5BB, and Harmony on flavan-3-ol concentrations. Contrarily, a recent study showed that ‘Merlot’ grafted onto SO4, 101-14, and 110R presented higher concentrations of proanthocyanidins in skins in one growing season. The differences in the rootstock effects on flavan-3-ols may be affected by scion cultivars and climatic differences, and further studies are necessary to understand this [31]. Notably, in this study, Ganzin 1 slightly reduced almost all the individual flavan-3-ol concentrations, indicating that Ganzin 1 tended to inhibit the accumulation of flavan-3-ols in grape skins. Interestingly, all the rootstocks except 110R imparted negative effects on epicatechin-3-*O*-galate concentrations and proportions; besides, significant decreases were observed in 2016 (Supplementary Figure S2). Therefore, we speculate that the accumulation of epicatechin-3-*O*-galate may be responsive to the graft. It was reported that catechin concentrations significantly differed among different grafted ‘Thompson Seedless’ vines [7]. By contrast, in this study, rootstocks had no marked effects on catechin concentrations and proportions, which may be ascribed to the scion cultivar differences.

**Figure 2:**
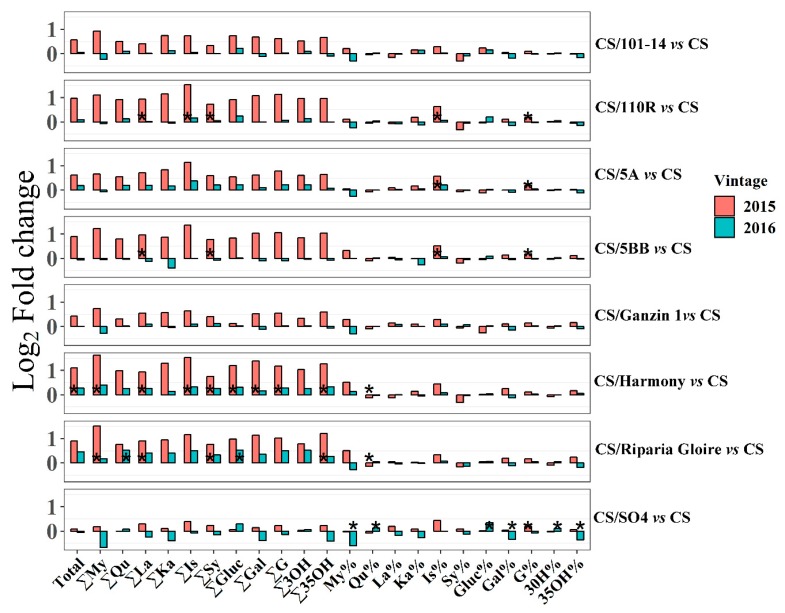
Effects of each rootstock on flavonols in two seasons (2015–2016). Each data represent the log2 fold change of flavonol concentrations/proportions in berries on each grafted vine relative to those on own-rooted vines. ‘Total’, the concentration of total flavonols; ‘∑’, the total concentration of different fractions of flavonols; ‘%’, the proportions of different fractions of flavonols; ‘My’, myricetin; ‘Qu’, Quercetin; ‘La’, laricitrin; ‘Ka’, kaempferol; ‘IS’, isohamnetin; ‘Sy’, syringetin; ‘Gluc’, flavonols in glucuronide form; ‘Gal’, flavonols in galactoside; ‘G’, flavonols in glucoside form; ‘3OH’, 3′-hydroxylated flavonols; ‘35OH’, 3′5′-hydroxylated flavonols. The asterisk * in each bar indicates significant differences between grafted and own-rooted vines in the same season at the basis of students’ *t*-test at *p* < 0.05.

In this study, flavonol concentrations and the proportions were not affected by the rootstocks, except Harmony (Figure 2). Quercetin, as the most abundant flavonol in ‘Thompson Seedless’, was also not statistically affected by different rootstocks [7]. Notably, 5A, Harmony, and Riparia Gloire increased the concentrations of all the individual flavonols in the two seasons, even though increases were not significant (Figure 2). These observations suggested that 5A, Harmony, and Riparia Gloire tended to promote flavonol biosynthesis.

**Figure 3:**
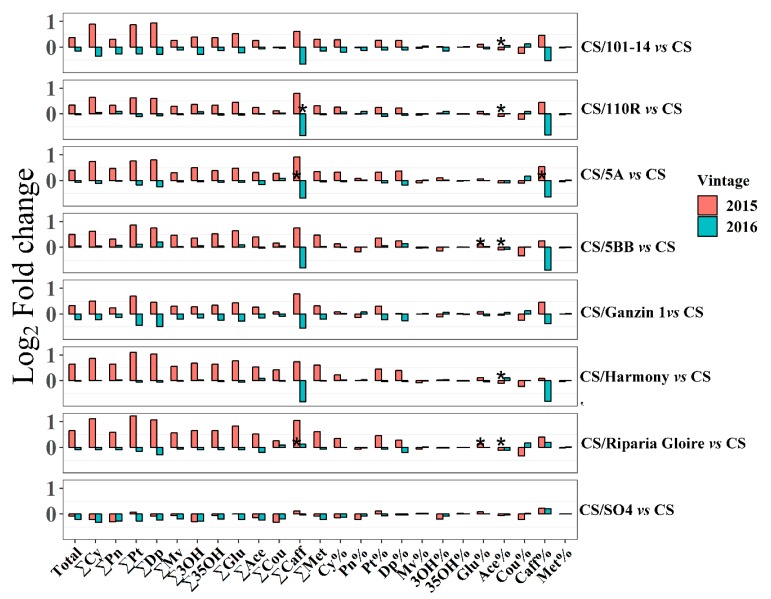
Effects of each rootstock on anthocyanins in two seasons (2015–2016). Each data represent the log2 fold change of anthocyanin content/proportion in berries on each grafted vine relative to those on own-rooted vines. ‘Total’, the concentration of total anthocyanins; ‘∑’, the total concentration of different fractions of anthocyanins; ‘%’, the proportions of different fractions of anthocyanins; ‘Cy’, cyanidin; ‘Pn’, peonidin; ‘Pt’, petunidin; ‘Dp’, delphinidin; ‘Mv’, malvidin; ‘Glu’, anthocyanins in glucoside form; ‘Ace’, acetylated anthocyanins; ‘Cou’, coumarylated anthocyanins; ‘Caff’, caffeoylated anthocyanins; ‘Met’, methoxylated anthocyanins; ‘3OH’, 3′-hydroxylated anthocyanins; ‘35OH’, 3′5′-hydroxylated anthocyanins. The asterisk * in each bar indicates significant differences between grafted and own-rooted vines in the same season at the basis of student’s *t*-test at *p* < 0.05.

In agreement with previous studies [4,11,30], rootstocks did not significantly affect anthocyanin concentrations in mature berries in this study (Figure 3). Nonetheless, few and minor differences in anthocyanin concentrations among grafted and own-rooted vines were found. Generally, the effects of grafted vines, except CS/SO4, on anthocyanin concentrations depended on seasons in this study. In 2015, except for SO4, all the rootstocks showed slight positive effects on anthocyanin accumulation. Notably, ‘Merlot’ and ‘Shiraz’ grafted onto 110R both possessed higher concentrations of anthocyanins than ungrafted grapevines in the previous studies [31,32]. However, in 2016, anthocyanin concentrations were slightly reduced by rootstocks, except 5BB and 110R, which exhibited a negligible influence. Notably, SO4 decreased most of the individual anthocyanin concentrations in two concessive seasons, indicating that SO4 tended to inhibit anthocyanin accumulation (Figure 3). Interestingly, SO4 showed the highest vine vigor (pruning weight) and tended to retard berry ripening, as mentioned above (Table 1 and Table 2). Reductions in anthocyanins induced by SO4 may be attributed to the differences in vine vigor between CS/SO4 and CS, which can alter the sunlight exposure accepted by grape berries [10]. In addition, sugar can regulate the expression of the genes encoding key enzymes in the anthocyanin biosynthesis pathway [33]. Therefore, the lower anthocyanin concentrations may have resulted from the lower amount of sugar in CS/SO4 in comparison with CS. In contrast, a recent study reported that SO4 led to ‘Merlot’ grapes with higher concentrations of anthocyanins in one season, which was attributed to the susceptibility to the biotic stress [31]. Regarding the proportions of different anthocyanin fractions, with few exceptions, there were no significant differences between grafted and own-rooted vines. 5BB and Riparia Gloire both statistically reduced the acetylated anthocyanin proportions. Besides, higher proportions of caffeoylated anthocyanins were observed in berries on CS/Riparia Gloire and CS/SO4 than in those on CS across the two seasons.

### 2.5. Effects of Rootstocks on Berry Volatile Compounds

There were 99 volatile compounds identified in grape berries during grape development in the two seasons (Supplementary Table S2). The O2PLS-DA (bidirectional orthogonal partial least squares discriminant analysis) strategy was applied to each rootstock and own-rooted vines (eight comparisons) in order to overcome the season effects and solely investigate the influence of each rootstock on volatile metabolites. In each O2PLS-DA model, a seven-fold internal cross-validation was conducted to validate the reliability of the model. The *R^2^Y* and *Q^2^Y* obtained from cross-validation measure the goodness of fit and the predictive ability of the O2PLS-DA model, respectively. Besides, a *p*-value was calculated in the cross-validation procedure (*p*_CV-ANOVA_) to estimate the significance of the O2PLS-DA model. Quality parameters of *Q^2^Y* above 0.5 and *p*_CV-ANOVA_ below 0.01 were set as thresholds to identify acceptable models. In addition, 500 permutations were performed to avoid overfitting of the O2PLS-DA model. Variable importance in projection (VIP) ranks the contribution of each variable to the O2PLS-DA model, and variables with VIP > 1.0 were considered as having the highest discrimination potential. Statistical differences in concentrations of volatile compounds between each rootstock and CS were determined according to the student’s *t*-test (*p* < 0.05) at each developmental stage. Through these criteria, those volatile compounds being consistently affected by rootstocks during development or at a certain developmental stage in the two seasons were characterized as volatile biomarkers.

Obtained O2PLS-DA models, except CS/Harmony vs. CS and CS/5A vs. CS, showed the clear separation between the grafted and own-rooted vines (Supplementary Figure S3). Values of *R^2^Y*, *Q^2^Y*, and *p*_CV-ANOVA_ of these O2PLS-DA models indicated excellent fit and predictive abilities, as well as the high significance of models (Table 3). In addition, 500 permutation tests of each O2PLS-DA model demonstrated that the predictive ability of the original model (*R^2^Y*, *Q^2^Y*) was higher compared to the permutated model (Supplementary Figure S3). Therefore, these O2PLS-DA models were reliable for discovering aromatic biomarkers of the grafted vines. Through O2PLS-DA analysis, CS/Harmony and CS/5A were not discriminated from own-rooted vines using volatile compounds, suggesting that CS/Harmony and CS/5A had no obvious influence on the accumulation of volatile compounds in this study.

**Figure 4:**
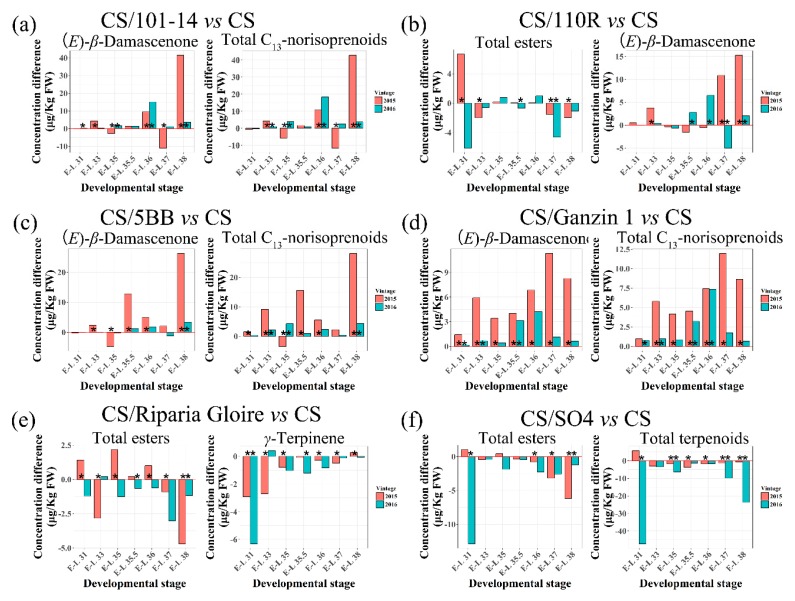
Effects of 101-14 (**a**), 110R (**b**), 5BB (**c**), Ganzin 1 (**d**), Riparia Gloire (**e**), and SO4 (**f**) on the volatile biomarkers in two seasons (2015–2016). Each data represent the concentration difference of volatile biomarkers between each grafted and own-rooted vines. The asterisk * in each bar indicates significant differences between grafted and own-rooted vines in the same season at the basis of student’s *t*-test at *p* < 0.05.

Through O2PLS-DA analysis, 26 volatile compounds with VIP > 1.0 were characterized to separate CS/101-14 from CS (Supplementary Table S3). Among these compounds, 101-14 had a positive influence on benzyl alcohol concentrations from E-L 31 stage until E-L 37, especially at E-L 35.5 and 36 stage, when 101-14 increased the concentration of benzyl alcohol significantly more than CS. At harvest, 101-14 slightly increased the concentration of benzyl alcohol in 2015, while the reverse was observed in 2016. Notably, 101-14 statistically enhanced (*E*)-*β*-damascenone concentrations in mature grape berries in two consecutive seasons. Since (*E*)-*β*-damascenone was the most abundant C_13_-norisoprenoid in grape berries, significantly higher concentrations of total C_13_-norisoprenoids were also observed in CS/101-14 compared to those in CS at harvest (Figure 4a).

There were 23 biomarker volatile compounds identified through O2PLS-DA to separate CS/110R from CS (Supplementary Table S3). Among these compounds, 110R had a negative influence on the concentrations of total esters, which were lower in CS/110R than those in CS at E-L 37 and 38 stage. In addition, despite the fact that 110R had an inconsistent influence on the concentrations of (*E*)-*β*-damascenone and d-limonene before harvest in the two seasons, it significantly increased the concentrations of (*E*)-*β*-damascenone and d-limonene in mature grape berries (Figure 4b).

The O2PLS-DA analysis revealed that 30 volatile compounds were responsible for the separation between CS/5BB and CS (Supplementary Table S3). 5BB showed a consistent positive influence on the accumulation of benzeneacetaldehyde, (*E*)-*β*-damascenone, total C_13_-norisoprenoids, and nerol oxide in the two seasons (Figure 4c).

With the first filtering of VIP > 1.0 in the O2PLS-DA model of CS/SO4 vs. CS, 24 volatile compounds were screened (Supplementary Table S3). Among these compounds, total esters and *γ*-terpinene were consistently affected by SO4. Particularly, SO4 tended to inhibit the accumulation of total esters after E-L 36 stage. The concentrations of total esters in CS/SO4 were statistically lower than those in CS at harvest in the two seasons (Figure 4d). In agreement with our results, Jin et al. [19] also reported that SO4 caused a reduction in ester content compared to own-rooted ‘Summer Black’. In addition, despite the fact that SO4 played a negative role in the accumulation of *γ*-terpinene and *α*-terpineol before harvest, no significant differences in the concentrations of *γ*-terpinene and *α*-terpineol between CS/SO4 and CS were found at harvest. The concentrations of total terpenoids were decreased by SO4 after E-L 31 stage; besides, significant decreases were found at harvest (Figure 4d).

The O2PLS-DA model of CS/Ganzin 1 vs. CS revealed that 22 volatile compounds highly contributed to the separation between CS/Ganzin 1 and CS (Supplementary Table S3). Through screening those compounds consistently impacted by CS/Ganzin 1 in the two seasons, the accumulation of (*E*)-*β*-damascenone and total C_13_-norisoprenoid was promoted by CS/Ganzin 1 (Figure 4e).

In the O2PLS-DA model of CS/Riparia Gloire vs. CS, 23 volatile compounds with VIP > 1.0 were filtered (Supplementary Table S3). Typically, the concentrations of (*Z*)-2-penten-1-ol in CS/Riparia Gloire remained lower compared to CS after E-L 36 stage. Statistically lower (*Z*)-2-penten-1-ol concentrations were observed at E-L 36 and 37 stage in 2015. Besides, Riparia Gloire significantly decreased the concentrations of total esters at harvest in the two seasons. Riparia Gloire inhibited the accumulation of *γ*-terpinene prior to harvest, except at E-L 33 stage in 2016. Notably, *γ*-terpinene concentrations were significantly lower in CS/Riparia Gloire in comparison to those in CS at E-L 31 stage. Inversely, at harvest, Riparia Gloire markedly increased the concentration of *γ*-terpinene in 2015 (Figure 4f).

Overall, 110R, Riparia Gloire, and SO4 induced reductions in the concentrations of total esters in mature berries. Besides, there were decreases in terpenoids, especially *γ*-terpinene and *α*-terpineol, in CS/SO4 compared to CS. Interestingly, 101-14, Ganzin 1, 110R, and 5BB led to increases in the concentrations of C_13_-norisoprenoids at harvest. Esters and terpenes both greatly contribute to the fruity and floral aromas. In addition, C_13_-norisoprenoids are characterized as important volatile compounds due to their low olfactory thresholds and pleasant smell. Therefore, with respect to the grape aroma profiles, SO4 may induce adverse effects, whereas CS/101-14, Ganzin 1, and 5BB can impart a positive influence.

## 3. Materials and Methods

### 3.1. Vineyard, Experiment Design, and Vine Management

The experimental vineyard is located at Shangzhuang experiment station (40°14′N, 116°20′E, 49 m altitude) of China Agricultural University, Beijing, China. The grape scion used in the field experiment was *Vitis vinifera* L. cv Cabernet Sauvignon clone 685, grafted on 101-14 (*Vitis riparia* × *Vitis rupestris*), 110R (*Vitis berlandieri* × *Vitis rupestris*), 5A (*Vitis berlandieri* × *Vitis riparia*), 5BB (*Vitis berlandieri* × *Vitis riparia*), Ganzin 1 (*Vitis vinifera* × *Vitis rupestris*), Harmony (1613 C × Dog ridge), Riparia Gloire (*Vitis riparia*), and SO4 (*Vitis berlandieri* × *Vitis riparia*). Performance of grafted vines and own-rooted vines was evaluated over two consecutive growing seasons (2015–2016). These vines were planted in 2011, spaced at 2.9 × 0.9 m with rows orientated south to north. In addition, a modified vertical shoot positioning (M-VSP) training system [34] was used in the vineyard, and this system was spur-pruned and retained 12–15 nodes per linear meter. The soils at the vineyard belonged to ‘loam’ soil (Supplementary Figure S1), and contained 9.64 g/kg organic matter. Besides, the soils’ electrical conductivity ranged from 0.17 to 0.21 mS/cm, belonging to non-saline. The soil pH was 8.08, and the soil cation exchange capacity was approximately 13.36 cmol/kg. Furrow irrigation was applied in this vineyard. Pest and nutrition management was conducted in accordance with the local industry standards. In addition, the meteorological data (mean monthly temperature, sunshine duration, and rainfall) of this vineyard during grape development were provided by the China Meteorological Data Sharing Service System (http://cdc.cma.gov.cn/).

### 3.2. Experiment Design and Sample Collection

This experiment was arranged in a randomized block design with three replicates of each graft combination. Each replicate consisted of ten vines. There were two buffer rows of own-rooted vines on either side of the experimental vineyard. Grape berries were sampled corresponding to seven phenological stages. Besides, the authors arbitrarily assigned the phenological stages to the decimal E-L stages [35]: (1) E-L 31 (pea-size berries); (2) E-L 33 (green berries); (3) E-L 35 (the onset of veraison); (4) E-L 35.5 (approximately 50% colored berries); (5) E-L 36 (berries reaching the medium maturity); (6) E-L 37 (berries not quite ripe); and (7) E-L 38 (harvest). No obvious differences in phenological stages among different grafted vines and own-rooted vines were observed in the two seasons. The flowering date of vines was 1st June and 21st May, respectively, in 2015 and 2016. The harvest was 15th October and 25th September, respectively, in 2015 and 2016. At each sampling point, 300 berries were randomly collected from both the sunny and shady side of clusters for each replicate. Physiochemical parameters of 100 berries were determined immediately after sampling, and the rest of the samples were frozen immediately in liquid nitrogen and stored at −80 °C.

### 3.3. Measurement of Vine Growth Parameters

For yield assessment, five vines with a similar vigor per replicate were randomly chosen at harvest. Cluster numbers per shoot and shoot numbers per vine were surveyed on these vines. Fifteen randomly sampled clusters per replicate were weighed to calculate the average cluster weight. Estimated yield per vine was obtained by multiplying the average cluster weight by the average cluster numbers per shoot and the average shoot numbers per vine. Pruning weight of the selected vines was recorded during winter pruning. In addition, after harvest, scion and rootstock diameters were measured 5 cm above and 5 cm below the graft union, respectively, using a digital caliper.

### 3.4. Determinations of Berry Physiochemical Parameter

A subsample of 100 berries was weighted, and then manually pressed. The must was centrifuged for 5 min at 8000 rpm, and the supernatant was determined for titratable acidity, pH, and total soluble solids (TSS), according to the method described by Wang et al. [34]. Determination of titratable acidity was conducted by titration with NaOH (0.05 M) to the end point of pH 8.2. Besides, titratable acidity was expressed as tartaric aid equivalent according to the National Standard of the People’s Republic of China (GB/T15308-2006, 2006). The Mettler Toledo FE20 Desktop pH Meter (Mettler, Toledo, Switzerland) was used for measuring the juice pH. Juice TSS was determined using the PAL-1 digital hand-held refractometer (Atago, Tokyo, Japan).

### 3.5. The Extraction of Flavonoids

Mature berry skins were manually peeled off in frozen status. Then, the skin was ground into powder, which was freeze-dried at −40 °C. The extraction of flavan-3-ols was consistent with the method described by Liang et al. [36]. The procedure of extracting free flavan-3-ol monomers was as follows: 0.100 g dried skin powder was mixed with 1.0 mL acetone/H_2_O (7:3, *v*/*v*) and 0.005 g ascorbic acid. The mixture was shaken for 15 min and then centrifuged for 15 min at 8000 rpm. The extraction of residue was conducted twice. All the supernatants were pooled, and 400 μL of the pooled supernatants was dried rapidly with a stream of dry nitrogen at 30 °C. The dried samples were dissolved in 200 μL acidified methanol with 1% (*v*/*v*) HCl and then neutralized with 200 μL aqueous sodium acetate (200 mM). The extraction of proanthocyanins is as follows: dried skin powder (0.100 g) was mixed with a solution (1.0 mL) of 0.3 M HCl in methanol with 50 g/L phloroglucinol and 5 g/L ascorbic acid. The mixture was incubated for 20 min at 50 °C and then combined with 1.0 mL aqueous sodium acetate (200 mM). The mixture was centrifuged for 15 min at 8,000 rpm. The residue was extracted twice. All the supernatants were collected and stored at ·40 °C until analysis. Flavonols and anthocyanins were extracted in accordance with the procedure reported by Downey et al. [37]. Dried skin powder (0.100 g) was macerated and sonicated in 50% (*v*/*v*) methanol in water (1.0 mL) for 20 min. The extraction was then conducted with centrifugation for 10 min at 12,000 rpm. The supernatant was collected and the residue was extracted twice. All the supernatants were pooled and stored at −40 °C.

### 3.6. High Performance Liquid Chromatography-Mass Spectrometry (HPLC-MS) Analysis of Flavonoids in Berry Skins

In accordance with the method described by Li et al. [38], flavan-3-ols in matured grape skin was analyzed using high performance liquid chromatography/triple-quadrupole tandem mass spectrometry (HPLC-QqQ-MS/MS). An Agilent 1200 series HPLC coupled with a Poroshell 120 EC-C18 column (150 × 2.1 mm, 2.7 μm) and diode array detector (DAD) and an Agilent 6410 QqQ instrument equipped with an electrospray ionization source were used. The analysis of flavonols in mature grape skin was performed, consistent with the method described by Sun et al. [39]. Agilent 1200 series HPLC-MSD trap VL linked simultaneously to a Zorbax EclipseXDB-C18 column (250 × 4.6 mm, 5 μm) and variable wavelength detector was used. Anthocyanins in mature grape skin were analyzed using Agilent 1100 series HPLC-MSD trap VL (Agilent, Santa Clara, CA, USA) coupled with a reversed phase Zorbax SB-C18 column (250 × 4 mm, 5 μm) and diode array detector (DAD) in accordance with the method reported by He et al. [40]. The content of flavan-3-ols was determined using (+)-catechin (C), (−)-epicatechin (EC), (−)-epigallocatechin (EGC), and (−)-epicatechin-3-*O*-galate (ECG), which were used as external standards. Flavonols and anthocyanins were quantified using quercetin-3-*O*-glucoside and malvidin-3-*O*-glucoside as external standards, respectively. All the concentrations of flavonoids were expressed as mg/kg berry fresh weight in this study.

### 3.7. Extraction of Volatile Compounds

Berries collected at seven developmental stages as described above were extracted for the analysis of volatile compounds. The extraction of volatile compounds followed the method described by Lan et al. [41]. For each replicate, sub-samples of 100 g de-seeded berries were grounded and blended with 1 g polyvinylpolypyrrolidone and 0.5 g d-gluconic acid lactone in liquid nitrogen. The blended powder was macerated at 4 °C for 4 h and then centrifuged at 8000 rpm at 4 °C for 10 min. Obtained clear juice was used for headspace solid phase microextraction (HS-SPME). HS-SPME was automatically conducted by a CTC-CombiPAL autosampler (CTC Analytics, Zwingen, Switzerland) equipped with a 2 cm DVB/CAR/PDMS 50/30 μm SPME fiber (Supelco, Bellefonete, PA., USA). Briefly, the mixture of 5 mL clear juice and 1 g NaCl, as well as 10 μL 4-methyl-2-pentanol (internal standard), was prepared in a 20 mL vial tightly capped with a PTFE-silicon septum. Samples were agitated at 500 rpm for 30 min at 40 °C, and then the SPME fiber, which was pre-conditioned at 250 °C for 1 h, was inserted into the headspace of the vial to absorb volatiles at 40 °C for 30 min. Afterwards, the SPME fiber was inserted into a gas chromatography injector for 8 min to thermally desorb volatile compounds.

### 3.8. Gas Chromatography-Mass Spectrometry (GC-MS) Analysis of Volatile Compounds

Volatile compounds were analyzed using Agilent 6890 GC coupled with Agilent 5973C MS. GC was equipped with an HP-INNOWAX capillary column (60 m × 0.25 mm, 0.25 μm, J&W Scientific, Folsom, CA, USA) to separate volatile compounds. Splitless mode was used, and the injection temperature was set at 250 °C. The flow rate of helium carrier gas was 1 mL/min. Temperature programmed chromatography was used: 50 °C for 1 min, increased to 220 °C at a rate of 3 °C /min, and held at 220 °C for 5 min. The temperature of the ion source and quadrupole was set at 250 °C and 150 °C, respectively. The full scan mode was employed to collect electron ionization mass data from *m*/*z* 30–350. The ionization voltage was set at 70 eV.

Mass spectrum analysis of the calculation of the retention indices (RI) of spectral components was conducted using the Automated MassSpectral Deconvolution and Identification System (AMDIS). Identification of volatile compounds was carried out through matching mass spectrum and RI with the reference standards in the NIST 11 MS database. Identified volatile compounds were quantified by an external standard method. A synthetic matrix was prepared by mixing 200 g/L glucose and 7 g/L tartaric acid in distilled water, and the pH the matrix was adjusted to 3.3 with 5 M NaOH solution. The standard solution was diluted into fifteen successive levels to obtain calibration curves. The concentrations of those volatile compounds without corresponding standards were estimated with equations of standards having the same functional group and/or similar numbers of carbon atoms. All the concentrations of volatile compounds were expressed as μg/kg berry fresh weight.

### 3.9. Chemicals

NaOH, HCl, methanol (analytical grade), phloroglucinol, and NaAc were supplied from Beijing Chemical Works. Acetonitrile, formic acid, and methanol (HPLC grade) were supplied from Fisher (Fairlawn, NJ, USA). C, EC, EGC, ECG, quercetin-3-*O*-glucoside, and malvidin-3-*O*-glucoside were purchased from Sigma-Aldrich (St. Louis MO, USA). Volatile standards listed in Supplementary Table S1, PVPP, and n-alkanes were purchased from Sigma-Aldrich (St. Louis, MO, USA).

### 3.10. Statistical Analysis

Differences in means of concentrations of compounds were determined by ANOVA by employing Duncan’s multiple range test at a level of *p* < 0.05 using the ‘agricolae’ package in R statistical environment (3.4.1). Supervised O2PLS-DA was carried out using SIMCA v14.1 (Umetrics, Umea, Sweden). Graphs were prepared using the ‘ggplot2′ package in R.

## 4. Conclusions

Generally, in the present study, rootstocks induced few or minor effects on Cabernet Sauvignon scion performance. Despite that, rootstocks had no significant effects on pruning weight and yield, and own-rooted vines produced the lowest pruning weight but the highest yield, indicating that vine balance was affected by rootstocks, which promoted the vegetative growth while inhibiting the productive growth. Although seasonal effects dominated the variations in berry physiochemical parameters, notably, 101-14, 5BB, and SO4 tended to retard berry ripening since these rootstocks reduced the total soluble solids while increasing the titratable acidity in berries. Ganzin 1 played a negative role in the accumulation of flavan-3-ols in skins. Interestingly, the concentrations and the proportions of epicatechin-3-*O*-galate in berry skins were negatively responsive to rootstocks, except 110R. 5A, Harmony, and Riparia Gloire showed tendencies for enhancing the flavonol level. In addition, SO4 led to reductions in most of the individual anthocyanin concentrations in the two seasons, showing adverse effects on the anthocyanin biosynthesis. With respect to volatile compounds in berries, lower concentrations of total esters were found in berries on vines grafted on 110R, Riparia Gloire, and SO4 compared to the berries on own-rooted vines. Additionally, 101-14, Ganzin 1, 110R, and 5BB led to increases in the concentrations of C_13_-norisoprenoids in mature berries. Overall, according to these results, selecting SO4 for grafting, considering the negative effects of SO4 on berry ripening and the accumulation of anthocyanin and volatile esters, was not recommended.

## Figures and Tables

**Table 1:** Effects of rootstocks on vine growth parameters in two seasons (2015–2016).

Source of Variation	Stock/Scion Diameter	Estimated Yield (kg/Vine)	Pruning Weight (kg/Vine)	Crop Load (Yield/Pruning Weight)
**Rootstock (R)**				
CS	1.00 a ^a^	4.27	1.26	4.26
CS/101-14	0.78 bc	3.38	1.41	3.02
CS/110R	0.75 bcd	3.52	1.38	3.08
CS/5A	0.77 bcd	4.10	1.40	4.15
CS/5BB	0.75 bcd	3.28	1.36	2.79
CS/Ganzin 1	0.69 cd	3.47	1.40	3.11
CS/Harmony	0.83 b	3.08	1.29	3.30
CS/Riparia Gloire	0.67 d	3.79	1.29	3.91
CS/SO4	0.71 cd	3.92	1.48	3.21
**Vintage (V)**				
2015	0.77	2.26 b	1.79 b	1.30 b
2016	0.77	5.03 a	0.93 a	5.55 a
**Significance** ^b^				
R × S	ns ^c^	ns	ns	ns

^a^ Values are reported as means of three biological replicates, and different letters within a row indicate significant differences among different vines or seasons on the basis of Duncan’s multiple range test at *p* < 0.05. ^b^ Significance of the interaction of rootstock and vintage. ^c^ ns represents not significant.

**Table 2:** Effects of rootstocks on physiochemical parameters of mature berries in two seasons (2015-2016).

Source of Variation	Berry Weight (g/100 Berries)	Total Soluble Solids (^o^Brix)	Titratable Acidity (g/L)	pH
**Rootstock (R)**				
CS	133.82 ^a^	20.10 ab	5.55	3.50
CS/101-14	133.64	19.73 ab	6.44	3.38
CS/110R	127.52	20.05 ab	5.48	3.39
CS/5A	144.87	19.35 ab	5.12	3.35
CS/5BB	137.27	19.65 ab	6.01	3.35
CS/Ganzin 1	153.33	20.1 ab	5.39	3.56
CS/Harmony	134.17	20.55 a	5.18	3.46
CS/Riparia Gloire	142.64	19.6 ab	4.68	3.42
CS/SO4	130.00	18.28 b	6.11	3.42
**Vintage (V)**				
2015	123.74 b	20.24 a	5.97	3.46
2016	147.62 a	19.08 b	5.39	3.38
**Significance** ^b^				
R × S	ns ^c^	ns	ns	ns

^a^ Values are reported as means of three biological replicates, and different letters within a row indicate significant differences among different vines or seasons on the basis of Duncan’s multiple range test at *p* < 0.05. ^b^ Significance of the interaction of rootstock and vintage. ^c^ ns represents not significant.

**Table 3:** Validation of O2PLS-DA models on volatile compounds in berries for the comparison of each grafted vine and own-rooted vine.

O2PLS-DA Models	Components ^a^	*R^2^X* (cum)	*R^2^Y* (cum)	*Q^2^* (cum)	*p_CV-ANOVA_*	500 Permutation Tests	
						*R^2^Y* Intercept	*Q^2^Y* Intercept
CS/101-14 vs. CS	1 + 6	0.94	0.78	0.61	4.31 × 10^−5^	0.39	−0.67
CS/110R vs. CS	1 + 5	0.91	0.86	0.72	5.07 × 10^−10^	0.36	−0.63
CS/5A vs. CS	1 + 2	0.92	0.52	0.38	0.09	0.20	−0.29
CS/5BB vs. CS	1 + 7	0.92	0.90	0.81	3.35 × 10^−13^	0.43	−0.83
CS/Ganzin 1 vs. CS	1 + 6	0.93	0.84	0.74	1.00 × 10^−9^	0.40	−0.71
CS/Harmony vs. CS	1 + 2	0.93	0.51	0.32	0.28	0.21	−0.30
CS/Riparia Gloire vs. CS	1 + 8	0.93	0.91	0.68	4.03 × 10^−7^	0.48	−0.99
CS/SO4 vs. CS	1 + 6	0.94	0.94	0.87	1.46 × 10^−14^	0.41	−0.81

^a^ representative of numbers of predictive components plus orthogonal components.

## Supplementary Materials

Supplementary materials can be found at http://www.mdpi.com/1422-0067/20/2/401/s1.

## Abbreviations

O2PLS-DA	Bidirectional orthogonal partial least squares-discriminant analysis
VIP	Variable importance in projection
CS	Cabernet Sauvignon
M-VSP	Modified vertical shoot positioning
TSS	Total soluble solids
HPLC-MS	High performance liquid chromatography-mass spectrometry
C	(+)-Catechin
EC	(−)-Epicatechin
EGC	(−)-Epigallocatechin
ECG	(−)-Epicatechin-3-*O*-galate
HS-SPME	Headspace solid phase microextraction
GC-MS	Gas chromatography-mass spectrometry
RI	Retention indices
AMDIS	Automated MassSpectral Deconvolution and Identification System

## References

[1] Ezzahouani A., Williams L.E. (1995). The influence of rootstock on leaf water potential, yield, and berry composition of Ruby Seedless grapevines. Am. J. Enol. Vitic..

[2] Avenant E., Avenant J.H., Barnard R.O. (1997). The effect of three rootstock cultivars, potassium soil applications and foliar sprays on yield and quality of *Vitis vinifera* L. cv. Ronelle in South Africa. S. Afr. J. Enol. Vitic..

[3] Stevens R.M., Pech J.M., Gibberd M.R., Walker R.R., Jones J.A., Taylor J., Nicholas P.R. (2008). Effect of reduced irrigation on growth, yield, ripening rates and water relations of Chardonnay vines grafted to five rootstocks. Aust. J. Grape Wine Res..

[4] Koundouras S., Hatzidimitriou E., Karamolegkou M., Dimopoulou E., Kallithraka S., Tsialtas J.T., Zioziou E., Nikolaou N., Kotseridis Y. (2009). Irrigation and rootstock effects on the phenolic concentration and aroma potential of *Vitis vinifera* L. cv. Cabernet Sauvignon grapes. J. Agric. Food Chem..

[5] Wooldridge J., Louw P.J.E., Conradie W.J. (2010). Effects of rootstock on grapevine performance, petiole and must composition, and overall wine score of *Vitis vinifera* cv. Chardonnay and Pinot noir. S. Afr. J. Enol. Vitic..

[6] Chitarra W., Perrone I., Avanzato C.G., Minio A., Boccacci P., Santini D., Gilardi G., Siciliano I., Gullino M.L., Delledonne M. (2017). Grapevine grafting: Scion transcript profiling and defense-related metabolites induced by rootstocks. Front. Plant Sci..

[7] Jogaiah S., Oulkar D.P., Banerjee K., Sharma J., Patil A.G., Maske S.R., Somkuwar R.G. (2013). Biochemically induced variations during some phenological stages in Thompson Seedless grapevines grafted on different rootstocks. S. Afr. J. Enol. Vitic..

[8] Cookson S.J., Ollat N. (2013). Grafting with rootstocks induces extensive transcriptional re-programming in the shoot apical meristem of grapevine. BMC Plant Biol..

[9] Berdeja M., Hilbert G., Dai Z., Lafontaine M., Stoll M., Schultz H., Delrot S. (2014). Effect of water stress and rootstock genotype on Pinot Noir berry composition. Aust. J. Grape Wine Res..

[10] Nelson C.C., Kennedy J.A., Zhang Y., Kurtural S.K. (2016). Applied water and rootstocks affect productivity and anthocyanin composition of Zinfandel in central California. Am. J. Enol. Vitic..

[11] Keller M., Mills L.J., Harbertson J.F. (2012). Rootstock effects on deficit-irrigated winegrapes in a dry climate: Vigor, yield formation, and fruit ripening. Am. J. Enol. Vitic..

[12] Conradie W.J. (1983). Liming and choice of rootstocks as cultural techniques for vines in acid soils. S. Afr. J. Enol. Vitic..

[13] Wooldridge J., Olivier M.P. (2014). Effects of weathered soil parent materials on Merlot grapevines grafted onto 110 Richter and 101-14Mgt rootstocks. S. Afr. J. Enol. Vitic..

[14] Sommer K., Clingeleffer P., Ollat N. (1993). Effects of minimal pruning on grapevine canopy development, physiology and cropping level in both cool and warm climates. Wein-Wissenschaft.

[15] Benz M.J., Anderson M.M., Williams M.A., Wolpert J.A. (2007). Viticultural performance of three Malbec clones on two rootstocks in Oakville, Napa Valley, California. Am. J. Enol. Vitic..

[16] Pulko B., Vršič S., Valdhuber J. (2012). Influence of various rootstocks on the yield and grape composition of Sauvignon blanc. Czech J. Food Sci..

[17] Loureiro M.D., Moreno-Sanz P., García A., Fernández O., Fernández N., Suárez B. (2016). Influence of rootstock on the performance of the Albarin Negro minority grapevine cultivar. Sci. Hortic..

[18] Corso M., Vannozzi A., Ziliotto F., Zouine M., Maza E., Nicolato T., Vitulo N., Meggio F., Valle G., Bouzayen M. (2016). Grapevine rootstocks differentially affect the rate of ripening and modulate auxin-related genes in Cabernet Sauvignon berries. Front. Plant Sci..

[19] Jin Z.-X., Sun T.-Y., Sun H., Yue Q.-Y., Yao Y.-X. (2016). Modifications of ‘Summer Black’grape berry quality as affected by the different rootstocks. Sci. Hortic..

[20] Jin Z.X., Sun H., Sun T.Y., Wang Q.J., Yao Y.X. (2016). Modifications of ‘Gold Finger’ grape berry quality as affected by the different rootstocks. J. Agric. Food Chem..

[21] Warschefsky E.J., Klein L.L., Frank M.H., Chitwood D.H., Londo J.P., von Wettberg E.J.B., Miller A.J. (2016). Rootstocks: Diversity, domestication, and impacts on shoot phenotypes. Trends Plant Sci..

[22] Habran A., Commisso M., Helwi P., Hilbert G., Negri S., Ollat N., Gomès E., van Leeuwen C., Guzzo F., Delrot S. (2016). Roostocks/scion/nitrogen interactions affect secondary metabolism in the grape berry. Front. Plant Sci..

[23] Nuzzo V., Matthews M.A. (2006). Response of fruit growth and ripening to crop level in dry-farmed Cabernet Sauvignon on four rootstocks. Am. J. Enol. Vitic..

[24] Wolpert J.A., Smart D.R., Anderson M. (2005). Lower petiole potassium concentration at bloom in rootstocks with Vitis berlandieri genetic backgrounds. Am. J. Enol. Vitic..

[25] Koblet W., Candolfivasconcelos M.C., Zweifel W., Howell G.S. (1994). Influence of leaf removal, rootstock and trainning system on yield and fruit composition of Pinot Noir grapevines. Am. J. Enol. Vitic..

[26] Kodur S., Tisdall J.M., Clingeleffer P.R., Walker R.R. (2013). Regulation of berry quality parameters in ‘Shiraz’ grapevines through rootstocks (*Vitis*). Vitis.

[27] Suriano S., Alba V., Di Gennaro D., Suriano M.S., Savino M., Tarricone L. (2016). Genotype/rootstocks effect on the expression of anthocyanins and flavans in grapes and wines of Greco Nero n. (*Vitis vinifera* L.). Sci. Hortic..

[28] Garcia M., Gallego P., Daverède C., Ibrahim H. (2001). Effect of three rootstocks on grapevine (Vitis vinifera L.) cv. Negrette, grown hydroponically. I. Potassium, calcium and magnesium nutrition. S. Afr. J. Enol. Vitic..

[29] Brancadoro L., Valenti L., Reina A., Scienza A. (1994). Potassium content of grapevine during the vegetative period-the role of the rootstock. J. Plant Nutr..

[30] Harbertson J.F., Keller M. (2012). Rootstock effects on deficit-irrigated winegrapes in a dry climate: Grape and wine composition. Am. J. Enol. Vitic..

[31] Gastón G., Encarna G., Bautista-Ortín A.B., Garde-Cerdán T., Moreno-Simunovic Y., Martínez-Gil A.M. Rootstock effects on grape anthocyanins, skin and seed proanthocyanidins and wine color and phenolic compounds from *Vitis vinifera* L. Merlot grapevines. J. Sci. Food Agric..

[32] Olarte Mantilla S.M., Collins C., Iland P.G., Kidman C.M., Ristic R., Boss P.K., Jordans C., Bastian S.E.P. (2018). Shiraz (*Vitis vinifera* L.) berry and wine sensory profiles and composition are modulated by rootstocks. Am. J. Enol. Vitic..

[33] Gollop R., Farhi S., Perl A. (2001). Regulation of the leucoanthocyanidin dioxygenase gene expression in *Vitis vinifera*. Plant Sci..

[34] Wang Y., He Y.N., Chen W.K., He F., Chen W., Cai X.D., Duan C.Q., Wang J. (2018). Effects of cluster thinning on vine photosynthesis, berry ripeness and flavonoid composition of Cabernet Sauvignon. Food Chem..

[35] Coombe B. (1995). Growth stages of the grapevine: Adoption of a system for identifying grapevine growth stages. Aust. J. Grape Wine Res..

[36] Liang N.-N., He F., Pan Q.-H., Wang J., Reeves M.J., Duan C.-Q. (2012). Optimization of sample preparation and phloroglucinol analysis of Marselan grape skin proanthocyanidins using HPLC-DAD-ESI-MS/MS. S. Afr. J. Enol. Vitic..

[37] Downey M.O., Mazza M., Krstic M.P. (2007). Development of a stable extract for anthocyanins and flavonols from grape skin. Am. J. Enol. Vitic..

[38] Li S.-Y., He F., Zhu B.-Q., Wang J., Duan C.-Q. (2016). Comparison of phenolic and chromatic characteristics of dry red wines made from native Chinese grape species and *Vitis Vinifera*. Int. J. Food Prop..

[39] Sun R.-Z., Cheng G., Li Q., He Y.-N., Wang Y., Lan Y.-B., Li S.-Y., Zhu Y.-R., Song W.-F., Zhang X. (2017). Light-induced variation in phenolic compounds in Cabernet Sauvignon Grapes (*Vitis vinifera* L.) involves extensive transcriptome reprogramming of biosynthetic enzymes, transcription factors, and phytohormonal regulators. Front. Plant Sci..

[40] He J.-J., Liu Y.-X., Pan Q.-H., Cui X.-Y., Duan C.-Q. (2010). Different anthocyanin profiles of the skin and the pulp of Yan73 (Muscat Hamburg × Alicante Bouschet) grape berries. Molecules.

[41] Lan Y.-B., Qian X., Yang Z.-J., Xiang X.-F., Yang W.-X., Liu T., Zhu B.-Q., Pan Q.-H., Duan C.-Q. (2016). Striking changes in volatile profiles at sub-zero temperatures during over-ripening of ‘Beibinghong’grapes in Northeastern China. Food Chem..

