# Supplementary material for: Rootstock-Mediated Effects on Cabernet Sauvignon Performance: Vine Growth, Berry Ripening, Flavonoids, and Aromatic Profiles
Source: Int J Mol Sci. 2019 Jan 18;20(2):401. doi: 10.3390/ijms20020401 (PMC6358736; doi:10.3390/ijms20020401)
Supplement: Supplementary file 1 [file ijms-20-00401-s001.zip › Supplementary Figures.docx]

**
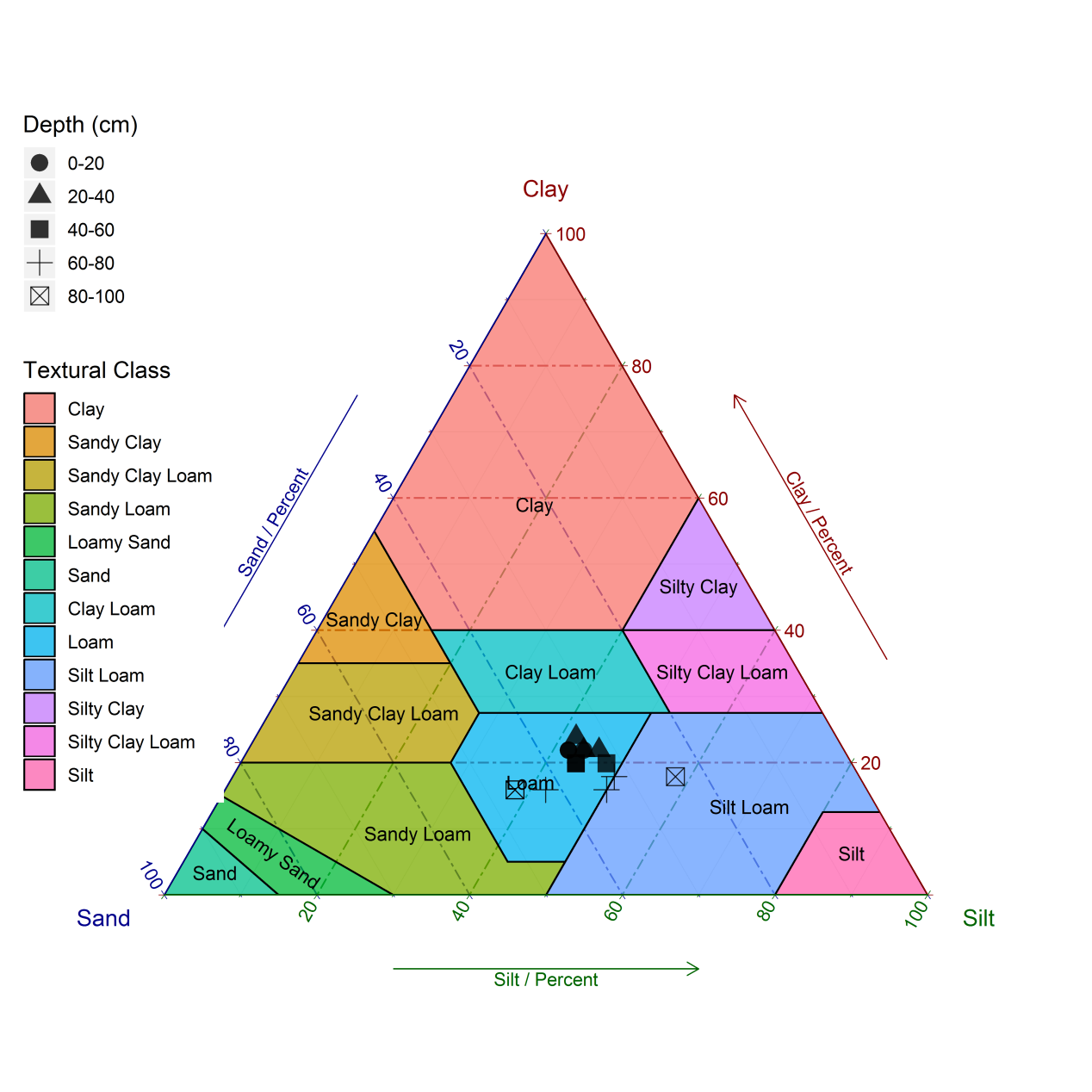
Supplementary Figure 1** Soil textural triangle for the soils of different depths in the vineyard. Different shapes correspond to the soils in different depths.

**
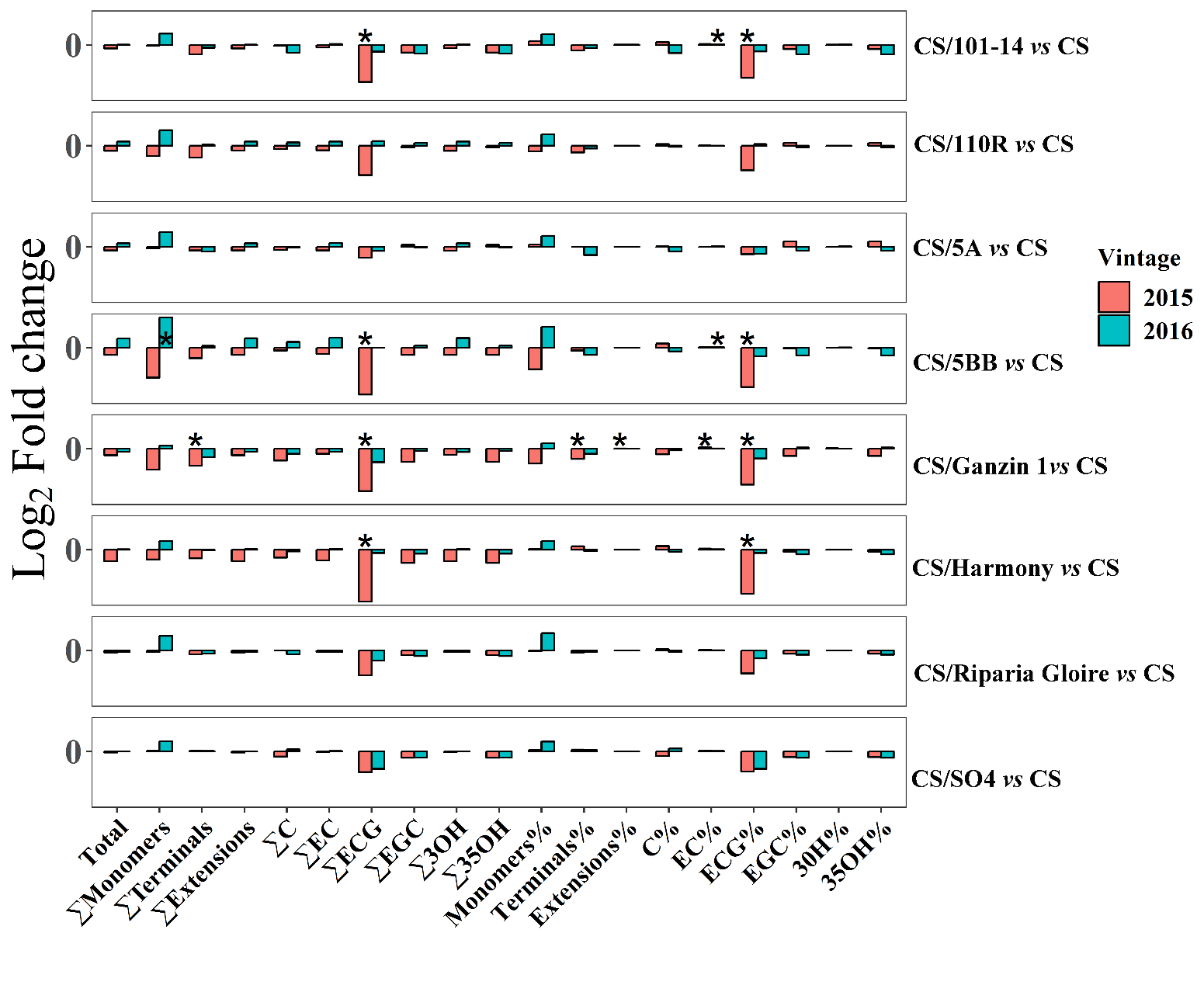
Supplementary Figure 2** Effects of each rootstock on flavan-3-ols in berry skins in two seasons (2015-2016). Each data represent the log2 fold change of flavan-3-ol content/proportion in berries on each grafted vines in relative to that on own-rooted vines. ‘Total’, total content of flavan-3-ols; ‘∑’, the total content of different fractions of flavan-3-ols; ‘%’, the proportions of different fractions of flavan-3-ols; ‘Monomers’, free flavan-3-ol monomers; ‘Terminals’, terminal flavan-3-ol subunits; ‘Extension’, extension flavan-3-ol subunits; ‘C’, catechin; ‘EC’, epicatechin; ‘ECG’, epicatechin-3-O-galate; ‘EGC’, epigallocatechin; ‘3OH’, 3’-hydroxylated flavan-3-ols; ‘35OH’, 3’5’-hydroxylated flavan-3-ols. The asterisk (*) in each bar indicated the significant differences between grafted and own-rooted vines in the same season on the basis of student’s *t*-test at *p* < 0.05.


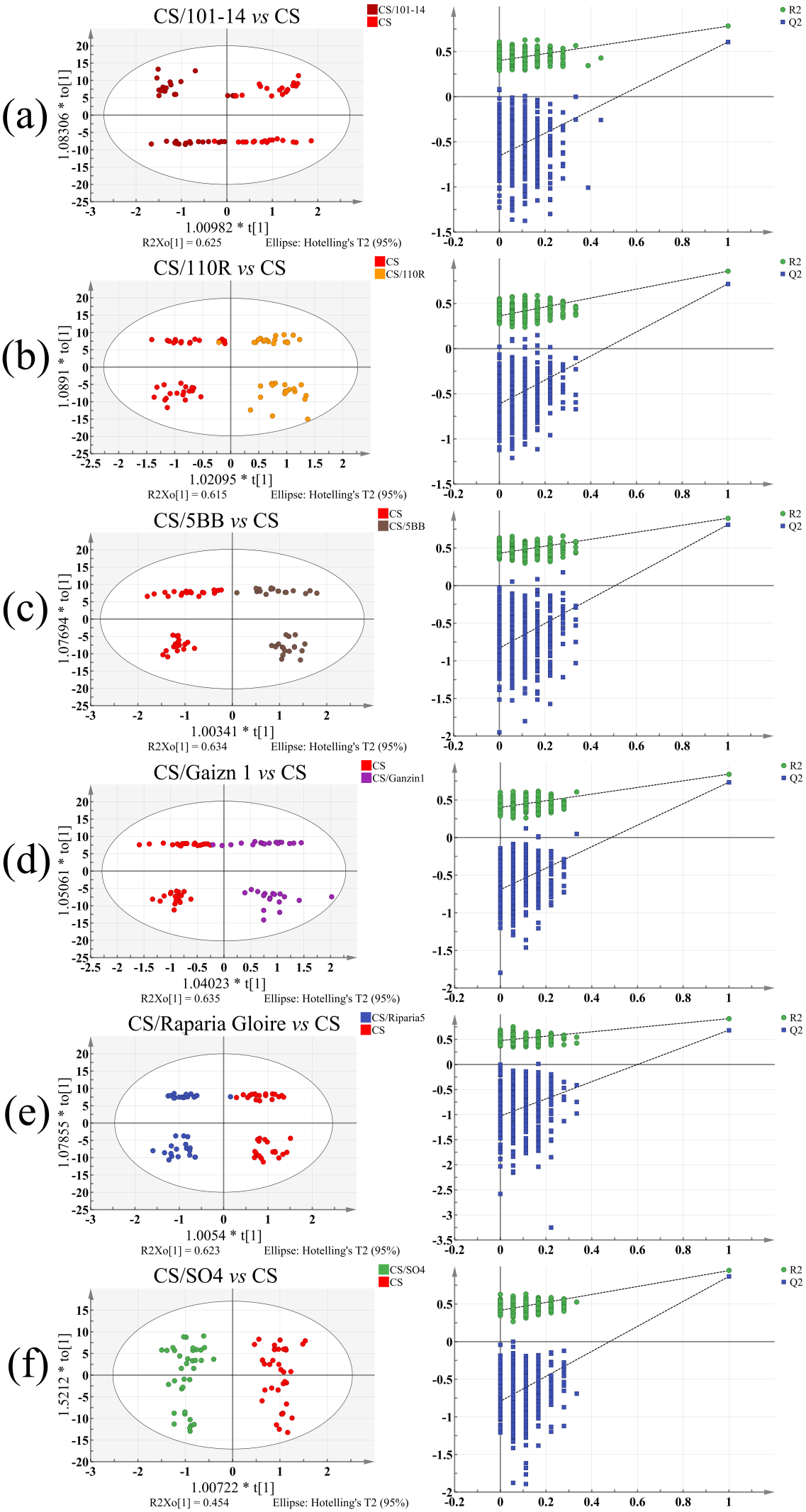


**Supplementary Figure 3** OPLS-DA plots (left column) showing the separation of each grafted vines ((a) CS/101-4; (b) CS/110R; (c) CS/5BB; (d) CS/Ganzin 1; (e) CS/Riparia Gloire; (f) CS/SO4 from the own-rooted vines based on the concentrations of volatile compounds in berries; The 500 permutation tests showing the validation of each O2PLS-DA model (right column).
